# Co-Delivery of Imiquimod and Plasmid DNA via an Amphiphilic pH-Responsive Star Polymer that Forms Unimolecular Micelles in Water

**DOI:** 10.3390/polym8110397

**Published:** 2016-11-16

**Authors:** Wenjing Lin, Na Yao, Hongru Li, Samuel Hanson, Wenqing Han, Chun Wang, Lijuan Zhang

**Affiliations:** 1 School of Chemistry and Chemical Engineering, South China University of Technology, Guangzhou 510640, China; lin.wenjing@mail.scut.edu.cn (W.L.); yao.na@mail.scut.edu.cn (N.Y.); 2 Department of Biomedical Engineering, University of Minnesota, Minneapolis, MN 55455, USA; lihongru@nankai.edu.cn (H.L.); hans3689@umn.edu (S.H.); hanxx144@umn.edu (W.H.); 3 State Key Laboratory of Medicinal Chemical Biology and Tianjin Key Laboratory of Molecular Drug Research, College of Pharmacy, Nankai University, Tianjin 300071, China

**Keywords:** co-delivery, pH-responsive, unimolecular micelles, immunotherapy

## Abstract

Dual functional unimolecular micelles based on a pH-responsive amphiphilic star polymer β-CD-(PLA-*b*-PDMAEMA-*b*-PEtOxMA)_21_ have been developed for the co-delivery of imiquimod and plasmid DNA to dendritic cells. The star polymer with well-defined triblock arms was synthesized by combining activator regenerated by electron-transfer atom-transfer radical polymerization with ring-opening polymerization. Dissipative particle dynamics simulation showed that core-mesophere-shell-type unimolecular micelles could be formed. Imiquimod-loaded micelles had a drug loading of 1.6 wt % and a larger average size (28 nm) than blank micelles (19 nm). The release of imiquimod in vitro was accelerated at the mildly acidic endolysosomal pH (5.0) in comparison to physiologic pH (7.4). Compared with blank micelles, a higher N*:*P ratio was required for imiquimod-loaded micelles to fully condense DNA into micelleplexes averaging 200–400 nm in size. In comparison to blank micelleplexes, imiquimod-loaded micelleplexes of the same N:P ratio displayed similar or slightly higher efficiency of gene transfection in a mouse dendritic cell line (DC2.4) without cytotoxicity. These results suggest that such pH-responsive unimolecular micelles formed by the well-defined amphiphilic star polymer may serve as promising nano-scale carriers for combined delivery of hydrophobic immunostimulatory drugs (such as imiquimod) and plasmid DNA with potential application in gene-based immunotherapy.

## 1. Introduction

With advances in genomics and proteomics, many new potential antigens have been identified and can be developed into subunit vaccines in the forms of recombinant proteins and synthetic peptides, as well as genetic vaccines based on DNA. Compared with traditional vaccines comprised of attenuated or deactivated pathogens, subunit- and gene-based antigens are much safer in humans but can be poorly immunogenic. Vaccine adjuvants are capable of enhancing immunogenicity of antigens either through exerting immunostimulatory effects or by altering the process of antigen delivery. Major categories of adjuvants include killed bacteria, bacterial components, aluminum salt, oil-based emulsions, polysaccharides, and liposomes [1,2,3,4,5]. Aluminum salt (Alum) is one of the most common adjuvants approved for human use, but its use is limited by suboptimal immunostimulatory capacity and difficulties in preparation and handling [6]. More specific and potent molecular adjuvants based on ligands of toll-like receptors (TLRs) have attracted much attention in recent years—some of the well-known ones include CpG oligodeoxynucleotides (TLR9 ligand), imidazoquinolines (TLR7/8 ligand), polyinosinic-polycytidylic acid (poly(I:C), TLR3 ligand), and monophosphoryl lipid A (MPL, TLR4 ligand), just to name a few [7]. On the other hand, these highly specific immunostimulatory molecules require new and better carriers to deliver them—along with antigens—to their respective cellular and molecular targets. Liposomes, emulsions, and polymeric nanoparticles are some of the current types of delivery systems that have shown much promise in enhancing the efficacy of molecular adjuvants and vaccines [8,9,10,11].

Imiquimod (IMQ) is a synthetic small-molecule immune response modifier (adjuvant). A member of the imidazoquinoline family, IMQ binds to TLR7 and stimulates the production of cytokines that activate the immune system to recognize and defend against viral infection and cancer [12,13]. A topical cream formulation containing 5% IMQ [14], marketed by 3M Pharmaceuticals (St. Paul, MN, USA) as Aldara, has been approved by the US Food and Drug Administration (FDA) to treat actinic keratosis, superficial basal cell carcinoma, and genital warts [15,16,17]. It is reported that topically-administered IMQ activates antigen-presenting Langerhan cells in the dermis [18], but a recent finding calls into question on whether the mechanism of action of Aldara is dependent on TLR7 [19], as the free drug is, highlighting the importance of the mode of drug delivery. Since IMQ is hydrophobic with its target TLR7 located inside the cell, it is logical to consider using polymeric microparticles [20] and nanoparticles [21,22] as delivery vehicles to improve aqueous solubility and facilitate intracellular transport of the drug. As demonstrated in recent reports by Trimaille and coworkers, using core-shell-type poly(d,l-lactide)-*b*-poly(*N*-acryloxysuccinimide-*co*-*N-*vinylpyrrolidone) micelles, encapsulation of IMQ into the hydrophobic core of micelles enhanced drug solubility in water and resulted in more potent stimulation of antigen-presenting dendritic cells in vitro [21,22].

The objective of this study is to develop unimolecular micelles as nanocarriers for combined delivery of IMQ and plasmid DNA to dendritic cells. In contrast to conventional micelles reported previously for IMQ delivery that are formed by self-assembly of multiple chains of linear block copolymer [21,22], unimolecular micelles are thermodynamically stable, because each micelle constitutes a single copy of an amphiphilic star polymer that maintains its structural integrity independent of polymer concentration, temperature, and pH [23,24,25,26]. At the same time, by controlling the length and composition of the star copolymer blocks, the properties of unimolecular micelles may be readily tunable to facilitate loading and release of IMQ in response to the intracellular pH environment of the endosome where stimulation of TLR7 occurs. Furthermore, cationic blocks can be introduced so that the unimolecular micelles also acquire the ability of delivering plasmid DNA in addition to IMQ. A number of cationic polymers are being investigated for DNA vaccine delivery to antigen-presenting cells (see examples [27,28,29,30]), however, polymeric carriers for combined delivery of plasmid DNA and hydrophobic immunostimulatory drugs (e.g., IMQ) have not been reported. Such dual functional nanocarriers would be useful in gene-based vaccination and immunotherapy, given the compelling bio-based rationale for the co-delivery approach [31]. Here we described the synthesis and characterization of such a dual functional nanocarrier and examined the influence of co-delivering IMQ on gene transfection efficiency in dendritic cells in vitro.

## 2. Materials and Methods

### 2.1. Materials

β-Cyclodextrin (β-CD, 99%, J&K Scientific Ltd., Beijing, China) was recrystallized from water, mixed in solvents (*n*-hexane/benzene) three times, and then dried at room temperature under reduced pressure. d,l-Lactide (d,l-LA, 99%, J&K Scientific Ltd, Beijing, China) was recrystallized from ethyl acetate three times and dried at room temperature under reduced pressure. 2-(Dimethylamino)ethyl methacrylate (DMAEMA, >98%, TCI, Tokyo, Japan) was purified by passing through a column filled with neutral alumina to remove inhibitor before use. Oligo(2-ethyl-2-oxazoline)methacrylate (EtOxMA) has been synthesized and reported in our previous article [32]. Triethylamine (TEA, 99%, Sigma-Aldrich, St. Louis, MO, USA), toluene (Sigma-Aldrich), tetrahydrofuran (THF, Sinopharm, Beijing, China), and anisole (Sinopharm) were dried over CaH_2_ and distilled under nitrogen. MgSO_4_, n-hexane, acetonitrile (J&K Scientific Ltd, Beijing, China), stannous octoate (Sn(Oct)_2_, Sinopharm), 2-bromoisobutyryl bromide (98%, Alfa Aesar, Haverhill, MA, USA), CuBr_2_ and 1,1,4,7,10,10-hexamethyltriethylenetetramine (HMTETA, 99%, Aldrich, St. Louis, MO, USA) were used as received. Ethidium bromide, 3-(4,5-Dimethylthiazol-2-yl)-2,5-diphenyltetrazolium bromide (MTT), and IMQ (98% pure) were purchased from Sigma (St. Louis, MO, USA). Dulbecco’s modified Eagle medium (DMEM), fetal bovine serum (FBS), penicillin, and streptomycin were all purchased from Invitrogen (Carlsbad, CA, USA).

### 2.2. Synthesis of β-CD-(PLA-b-PDMAEMA-b-PEtOxMA)_21_

The amphiphilic pH-responsive star copolymer was synthesized by combining activator regenerated by electron-transfer atom-transfer radical polymerization (ARGET ATRP) with ring opening polymerization (ROP) [32]. First, the synthesis of β-CD-(PLA-OH)_21_ by ROP of d,l-LA (5.50 g, 37.8 mmol) in anhydrous toluene (25 mL) was carried out using β-CD (681 mg, 0.6 mmol) as an initiator and Sn(Oct)_2_ (65 µL, 0.2 mmol) as a catalyst at 130 °C for 24 h. The macroinitiator β-CD-(PLA-Br)_21_ was then synthesized from β-CD-(PLA-OH)_21_ (5.50 g, 0.6 mmol) and TEA (3.34 mL, 24 mmol) with 2-bromoisobutyryl bromide (2.97 mL, 24 mmol) in anhydrous THF (100 mL) at room temperature. Sequentially, monomer DMAEMA (7.07 mL, 42 mmol) and macroinitiator β-CD-(PLA-Br)_21_ (4.2 g, 0.4 mmol) were introduced to produce β-CD-(PLA-*b*-PDMAEMA)_21_ in 20 mL anhydrous toluene at 70 °C. A small amount of CuBr_2_ catalyst (6.7 mg, 0.03 mmol) and ligand HMTETA (76 µL, 0.3 mmol) were used together with a sufficiently large excess of reducing agent Sn(Oct)_2_ (97 µL, 0.30 mmol). Finally, β-CD-(PLA-*b*-PDMAEMA-*b*-PEtOxMA)_21_ was polymerized in anhydrous anisole (20 mL) at 60 °C by adding EtOxMA (9.08 g, 11.34 mmol) and β-CD-(PLA-PDMAEMA-Br)_21_ (2.5 g, 0.06 mmol). The amount of CuBr_2_ catalyst and reducing agent Sn(Oct)_2_ was the same as β-CD-(PLA-*b*-PDMAEMA)_21_ polymerization with the exception of the ligand HMTETA (0.164 mL, 0.64 mmol). ^1^H NMR spectra were acquired in CDCl_3_ at 25 °C using a Bruker AVANCE ΙΙΙ 400 (Madison, WI, USA) operating at 400 MHz. ^1^H NMR of the final polymer product (CDCl_3_, *δ*, ppm): 5.17 (1H, C*H* of PLA), 4.05 (2H, –OC*H*_2_ of PDMAEMA), 3.44 (4H, N–C*H*_2_C*H*_2_ of PEtOxMA), 3.02 (3H, N–C*H*_3_ of PEtOxMA), 2.56 (2H, –C*H*_2_N of PDMAEMA), 2.30 (6H, –N(C*H*_3_)_2_ of PDMAEMA and PEtOxMA), 1.93 (6H, (C*H*_3_)_2_–Br), 1.70–1.90 (2H, –C*H*_2_CH_3_ of PDMAEMA and PEtOxMA), 1.55 (3H, CHC*H*_3_ of PLA), 0.81–1.10 (3H, –CH_2_C*H*_3_ of PDMAEMA and PEtOxMA).

### 2.3. Critical Micellar Concentration (CMC) Determination

Nile red was used as the fluorescent probe for CMC determination, starting from micellar solutions of varying concentrations ranging from 0.00025–20.0 mg/mL. To each sample, 20 μL of Nile red in acetone (3.0 × 10^−4^ M) was added to result in a final Nile red in water concentration of 1.5 × 10^−6^ M. After evaporating acetone overnight, the samples were equilibrated for three days. The fluorescence emission spectra were recorded between 525 and 750 nm with λ_ex_ = 550 nm.

### 2.4. Preparation and Characterization of Blank and IMQ-Loaded Micelles

The blank and IMQ-loaded micelles were prepared using the diafiltration method [33,34,35]. To prepare IMQ-loaded micelles, IMQ (8 mg) was dissolved in 10 mL of DMSO. The polymer (40 mg) was dissolved in another 10 mL of DMSO, combined with the IMQ/DMSO solution, and stirred for 4 h. The IMQ/polymer/DMSO solution was then transferred to a dialysis bag (MW cut-off: 1 kDa) and dialyzed against 2 L of deionized water for 24 h to remove the DMSO and free drug. The deionized water was changed every 2 h for the first 6 h and then replaced every 6 h. After dialysis, the micelles were filtered through a membrane filter with 0.45-μm pores to remove aggregated particles and then collected by freeze-drying. The dried micelle product, as white powder, was stored at −20 °C. Blank micelles were prepared in the same way except without IMQ.

The average size and polydispersity index (PDI) of micelles were measured by dynamic light scattering (DLS) using a ZetaPlus particle analyzer (Brookhaven Instruments Corp., Holtsville, NY, USA; 27 mW laser; 658 nm incident beam, 90° scattering angle) at a polymer concentration of 0.1 mg/mL. Morphology of micelles at the same concentration was examined by transmission electron microscopy (TEM) using a Hitachi H-7650 microscope (Hitachi, Tokyo, Japan) operating at 80 kV. To investigate IMQ solubility, UV spectra of IMQ-loaded micelles in the 300–350 nm range were recorded using a BioTek Synergy HT microplate reader (BioTek Instruments, Winooski, VT, USA). To quantify IMQ loading, 2 mg of IMQ-loaded micelles were dissolved in 2 mL of DMSO and then analyzed to record UV absorbance at 318 nm using the same plate reader. The background of blank micelles in DMSO was subtracted. The amount of IMQ was calculated using a calibration curve constructed from measurements of samples with known concentrations of IMQ.

Dissipative particle dynamics (DPD) simulation, based on the coarse-grained models, was employed to gain deeper insight into the formation and microstructure of unimolecular micelles through simulating an aqueous solution of polymer with the same concentration as experiment (0.1 mg/mL). The interaction parameters were calculated according to our previous method [32,36]. DPD simulations were conducted in the Mesocite module at 298.15 K in Materials Studio 5.0 (Accelrys Inc., San Diego, CA, USA). A cubic simulation box of 37 × 37 × 37 *r*_c_^3^ with periodic boundary condition was applied in all three directions. The integration time step was 0.05 ns and the number of simulation steps was 100,000.

### 2.5. In Vitro Release Kinetics of IMQ

Release kinetics of IMQ from micelles was measured at different pHs: 7.4 (physiological pH) and 5.0 (mildly acidic pH of the endolysosomal compartment of cells), and a procedure of measurement was adapted from a method previously used for quantifying the release of water-insoluble doxorubicin from micelles [37,38]. Briefly, IMQ-loaded micelles were suspended at 1 mg/mL in a 20 mM phosphate buffer (pH 7.4) or acetate buffer (pH 5.0). Both solutions were stirred in a shaker at 37 °C. Absorbance of each solution was measured at 318 nm using a microplate reader (Synergy HT, BioTek) at predetermined time points. Each experiment was performed three times.

### 2.6. Preparation and Analyses of Micelle-DNA Complexes (Micelleplexes)

A plasmid containing the gene of green fluorescence protein (GFP) and a cytomegalovirus (CMV) promoter (pEGFP-N1) was used for evaluating micelleplex properties and gene transfection. Micelleplexes of N:P ratios ranging from 1/4 to 30 were prepared by adding 15 μL of micelle solution in 20 mM N-2-hydroxyethylpiperazine-N-2-ethane sulfonic acid (HEPES) (pH 7.4) to 15 μL of DNA solution (0.2 μg/μL in 20 mM HEPES, pH 7.4), vortexed for 10 s, and incubated for 30 min at room temperature. Both blank and IMQ-loaded micelles were used to prepare micelleplexes with plasmid DNA using the same procedure.

Gel retardation assay of micelleplexes of various N:P ratios was carried out by electrophoresis on a 1.0% agarose gel containing 0.5 μg/mL of ethidium bromide (EB). Exclusion of EB due to micellplex formation was quantified by measuring the fluorescence intensity of the dye using a Bio-Tek Synergy HT plate reader with excitation wavelength of 530/25 nm and emission wavelength of 590/35 nm. The results were presented as normalized percentages with measurement of the naked DNA/EB solution as 100% and that of the EB solution without DNA as 0%. The average size and PDI of micelleplexes in 20 mM HEPES buffer at 25 °C were determined using the same ZetaPlus particle analyzer mentioned above. Micelleplexes with N:P ratios ranging from 1–40 were prepared as described above and diluted 20 times to a final volume of 2 mL in HEPES buffer before measurement.

### 2.7. MTT (3-(4,5-Dimethylthiazol-2-yl)-2,5-Diphenyltetrazolium Bromide) Assay

Murine DC2.4 cells (ATCC, Manassas, VA, USA) were seeded in a 96-well plate at a density of 1 × 10^4^ cells/well in 200 µL of complete DC2.4 media (low-glucose DMEM, 10% FBS, 10 mM HEPES, 100 U/mL penicillin/streptomycin) and allowed to grow for 24 h to reach 60%–70% confluence. Each well was then replaced with 200 µL of micelleplex solutions of various N:P ratios and the treated cells were incubated in 5% CO_2_ at 37 °C for 24 h. This test was performed in replicates of six wells and cell viability was calculated as previously described [28].

### 2.8. In Vitro Gene Expression

The procedure was adapted from a previous report [28]. DC2.4 cells were seeded into 12-well plates at 100,000 cells/well and cultured in 5% CO_2_ and 37 °C overnight. After removing the cell media, cells were washed by phosphate buffered saline (PBS) twice followed by adding DC2.4 media without serum. Cells were then transfected for 4 h with micelleplexes of GFP plasmid at various N:P ratios either with or without IMQ loaded. After discarding the media, cells were washed with PBS twice and cultured in serum-containing media for another 20 h. The cells were harvested by treating with trypsin-ethylenediaminetetraacetic acid (EDTA), dispersed in fluorescence-activated cell sorting (FACS) buffer (PBS containing 1% bovine serum albumin), and analyzed using a flow cytometer (Accuri C6, BD Biosciences, San Jose, CA, USA). DC2.4 cells were also transfected with micelleplexes of a luciferase plasmid under the same conditions to exclude the autofluorescence of cells. The software FlowJo (Ashland, OR, USA) was utilized to determine the percentage of GFP^+^ cells and mean fluorescence intensity (MFI). The gate was drawn based on the luciferase control where false positive frequency was restricted to below 0.2%.

### 2.9. Statistical Analysis

Statistical analysis was carried out using a two-sample Student’s *t*-test with unequal variance. *p* < 0.05 was considered statistically significant.

## 3. Results and Discussion

### 3.1. Rationale of Polymer Design

**Scheme 1:**
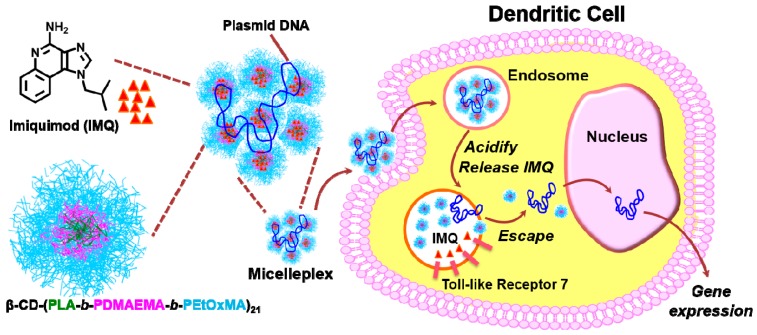
Schematic illustration of co-delivery of IMQ (red triangles) and plasmid DNA to dendritic cells via unimolecular micelles formed by the amphiphilic pH-responsive star polymer β-CD-(PLA-*b*-PDMAEMA-*b*-PEtOxMA)_21_.

The amphiphilic pH-responsive star block copolymer β-CD-(PLA-*b*-PDMAEMA-*b*-PEtOxMA)_21_ was designed to form core-mesophere-shell-type unimolecular micelles as nanocarriers for loading and intracellular delivery of IMQ and plasmid DNA. The star polymer had a β-CD core and 21 arms, each consisting of blocks of PLA, PDMAEMA, and PEtOxMA, in that order. The innermost PLA block formed the hydrophobic core of the unimolecular micelle, in which the poorly-soluble IMQ could be encapsulated. The outermost block of PEtOxMA brushes formed the hydrophilic shell for stabilization, and could avoid unspecific interactions with the protein [39,40,41]. The middle block of pH-responsive PDMAEMA contains tertiary amines that become more protonated and cationic at mildly acidic pH, was expected to facilitate release of IMQ inside the endolysosome of dendritic cells where TLR7, the molecular target of IMQ, is located. This property of the PDMAEMA block should also enable binding, condensation, and intracellular release of the anionic plasmid DNA that culminate in the transfection of dendritic cells. These hypotheses were illustrated in Scheme 1.

### 3.2. Synthesis and Characterization of the Star Block Copolymer

**Scheme 2:**
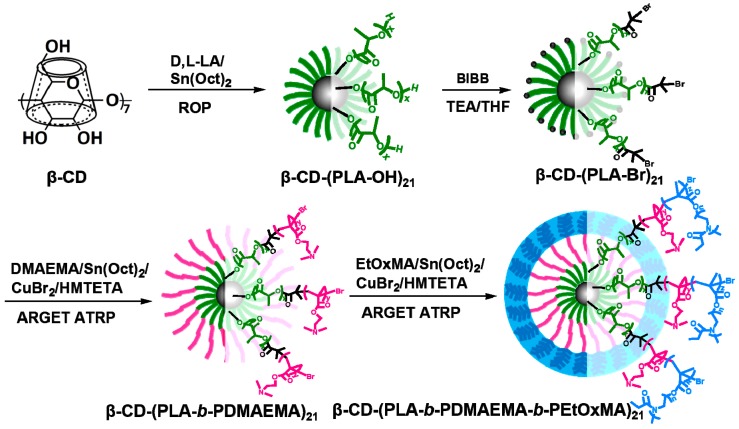
Schematic illustration of the synthesis of β-CD-(PLA-*b*-PDMAEMA-*b*-PEtOxMA)_21_.

Controlled living free radical polymerization techniques have been increasingly used in the synthesis of well-defined polymers for investigating structure-function relationship of drug and gene delivery systems [42,43,44,45,46]. We have reported recently multi-step synthesis of star block copolymers based unimolecular micelles as templates for in situ formation of gold nanoparticles [32]. Here, through combined use of ARGET ATRP and ROP, we have synthesized a new star block copolymer β-CD-(PLA-*b*-PDMAEMA-*b*-PEtOxMA)_21_, as shown in Scheme 2.

**Figure 1:**
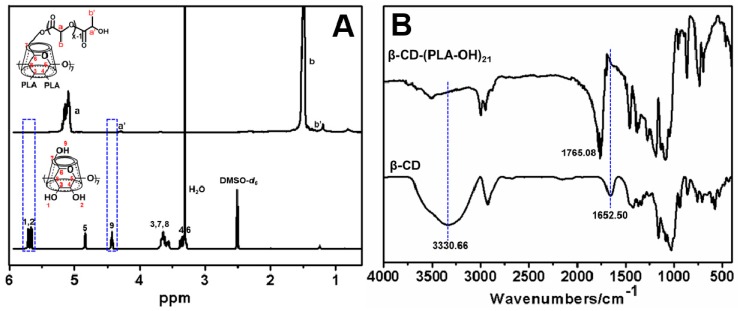
The complete conversion of 21 hydroxyl groups of pristine β-CD to PLA arms. (**A**) ^1^H NMR spectra of β-CD in DMSO-*d*_6_ and β-CD-(PCL-OH)_21_ in CDCl_3_; (**B**) FT IR spectra of β-CD and β-CD-(PCL-OH)_21_.

**Figure 2:**
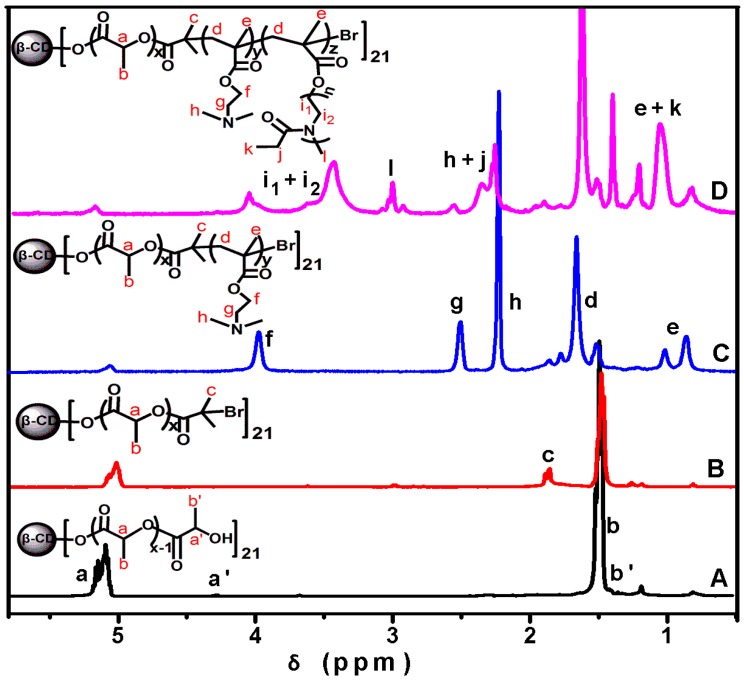
^1^H NMR spectra of β-CD-(PLA-OH)_21_ (**A**); β-CD-(PLA-Br)_21_ (**B**); β-CD-(PLA-*b*-PDMAEMA)_21_ (**C**); and β-CD-(PLA-*b*-PDMAEMA-*b*-PEtOxMA)_21_ (**D**) in CDCl_3_.

^1^H NMR spectra in Figure 1A displayed the characteristic proton peaks of PLA, accompanied by the disappearance of the peaks (4.43 and 5.66–5.71 ppm) from the hydroxyl groups of pristine β-CD, which confirmed the complete conversion of 21 hydroxyl groups of native β-CD to PLA arms. This observation was further supported by the disappearance of O–H stretching vibration (3330.66 cm^−1^) of β-CD after converting to β-CD-(PCL-OH)_21_ (carbonyl stretching vibration, 1765.08 cm^−1^) (Figure 1B). We then presented another ^1^H NMR spectra, which clearly showed characteristic chemical shifts of protons in the prepolymers and final polymer products (Figure 2). Through integrating the areas of characteristic peaks including the end-groups, the degree of polymerization (DP) of each block and molecular weight of the polymer product were estimated [47,48].

*DP*_PLA_ = *I*_a_/*I*_a’_ (1)

*DP*_PDMAEMA_ = *I*_f_/*I*_a_ × 1/2 (2)

*DP*_PEtOxMA_ = *I*_l_/*I*_a_ × 1/3 (3)

where *I*_a_, *I*_a’_, *I*_f_, and *I*_l_ are the intensity of the peaks at 5.17, 4.35, 4.05, and 3.02 ppm, respectively. Thus, the average molecular weight of the prepolymers and polymer product was obtained:

*M*_n β-CD-(PLA-OH)__21_ = *DP*_PLA_ × 72 + 1135 (4)

*M*_n β-CD-(PLA-Br)__21_ = *DP*_PLA_ × 72 + 4264 (5)

*M*_n__β-CD-(PLA-b-PDMAEMA)__21_ = (*DP*_PLA_ × 72 + *DP*_PDMAEMA_ × 157) + 4264 (6)

*M*_n__β-CD-(PLA-b-PDMAEMA-b-PEtOxMA__)21_ = (*DP*_PLA_ × 72 + *DP*_PDMAEMA_ × 157+ *DP*_PEtOxMA_ × 801) + 4264 (7)

where the molecular weights of β-CD, β-CD-(Br)_21_, d,l-LA, DMAEMA, and EtOxMA are 1135, 4264, 72, 157, and 801 g/mol, respectively. The DP of PLA, PDMAEMA, and PEtOxMA blocks were 6, 5 and 8, respectively, and the molecular weight values of β-CD-(PLA-OH)_21_, β-CD-(PLA-Br)_21_, β-CD-(PLA-*b*-PDMAEMA)_21_, and β-CD-(PLA-*b*-PDMAEMA-*b*-PEtOxMA)_21_ determined by ^1^H NMR were 10,207, 13,336, 29,821, and 164,389 g/mol, respectively, demonstrating that the star polymer with high purity has been successfully synthesized.

### 3.3. Simulation and Characterization of Unimolecular Micelles

**Figure 3:**
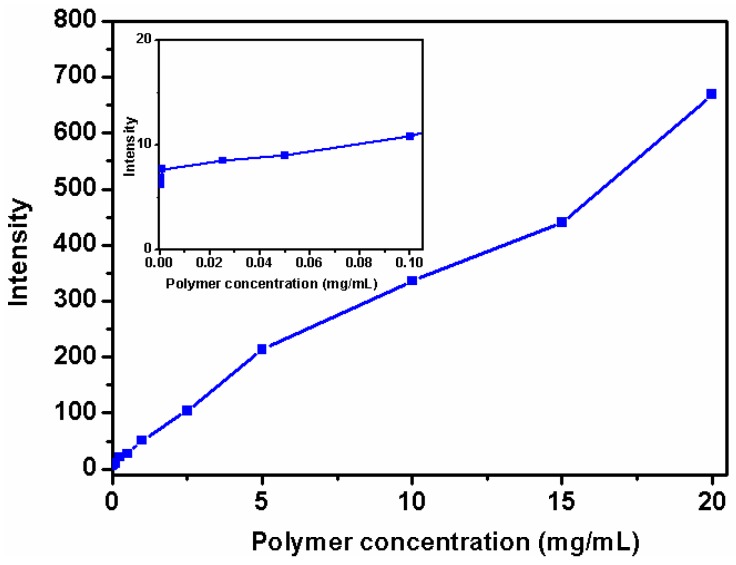
Intensity of the fluorescence emission of Nile red at 620 nm (λ_ex_ = 550 nm; slit widths: *E*x. 5 nm, *E*m. 5 nm) as a function of concentration of β-CD-(PLA-*b*-PDMAEMA-*b*-PEtOxMA)_21_ as well as an expanded view of the low concentration range (inset).

The experiment attempted for CMC determination was performed with Nile red as a fluorescent probe, which has low solubility in water and will be solubilized within the hydrophobic PLA core [25]. Figure 3 showed that the fluorescence emission intensity of Nile red at 620 nm increased with increasing star polymer concentration, even at extremely low polymer concentrations. No abrupt changes in fluorescence intensity were observed throughout the entire range of polymer concentration, indicating the absence of a CMC in water and, thus, confirming the existence of unimolecular micelles.

**Figure 4:**
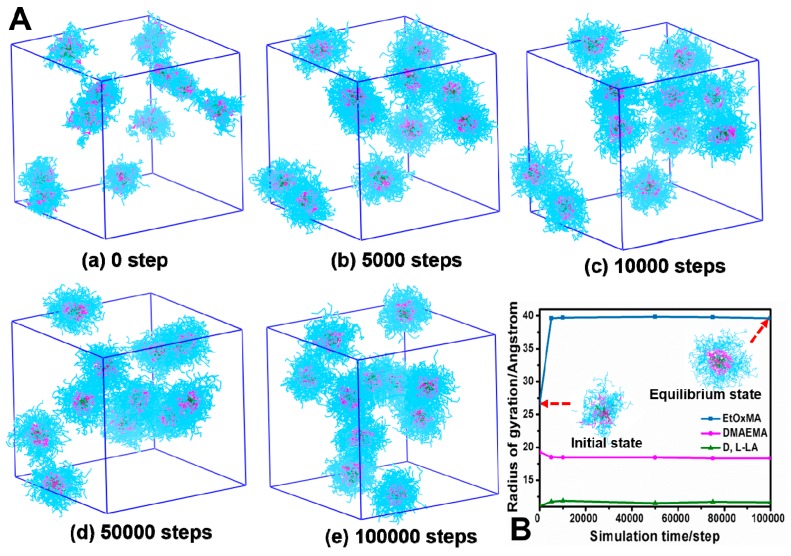
DPD simulation of β-CD-(PLA-*b*-PDMAEMA-*b*-PEtOxMA)_21_ at different simulation times at experimental concentration of 0.1 mg/mL. (**A**) Formation process. (**B**) Radius of gyration of EtOxMA, DMAEMA, and d,l-LA.

To gain insight on the solution behavior of the star block copolymer and formation of micelles, DPD simulation was conducted using the same polymer concentration as in actual experiments (0.1 mg/mL). The hydrophobic block, pH-responsive block, and hydrophilic block of the star polymer were shown in green, pink, and blue color, respectively, while water beads (grey color) were hidden for clarity. Shown in Figure 4A, the simulation started with 11 star polymer molecules dispersed randomly in the box. As the system moved toward equilibrium, 11 discrete micelles were observed. The number of micelles was the same of the number of polymer molecules, evidence that these were unimolecular micelles. With the elapsing time of simulation, the surface of the micelles became smoother and more spherical. The radius of gyration of EtOxMA increased gradually and then became stable, while the radius of gyration of DMAEMA and d,l-LA remained unchanged, which may due to the fact that the extension of long hydrophilic EtOxMA results in the mild increase of the micellar size (Figure 4B). The hydrophilic EtOxMA are of good compatibility with water and abundant quantity (one polymer molecule contained 1176 EtOxMA beads), while the number of both hydrophobic d,l-LA beads and the DMAEMA beads were 63 and 105, respectively. Therefore, the aggregation contributed by hydrophobic effect was avoided in our micelle system, which existed as core-mesophere-shell-type unimolecular micelles with PLA block as hydrophobic core, PDMAEMA as the middle layer, and dense PEtOxMA brushes as the hydrophilic shell to maintain colloidal stability in water.

**Figure 5:**
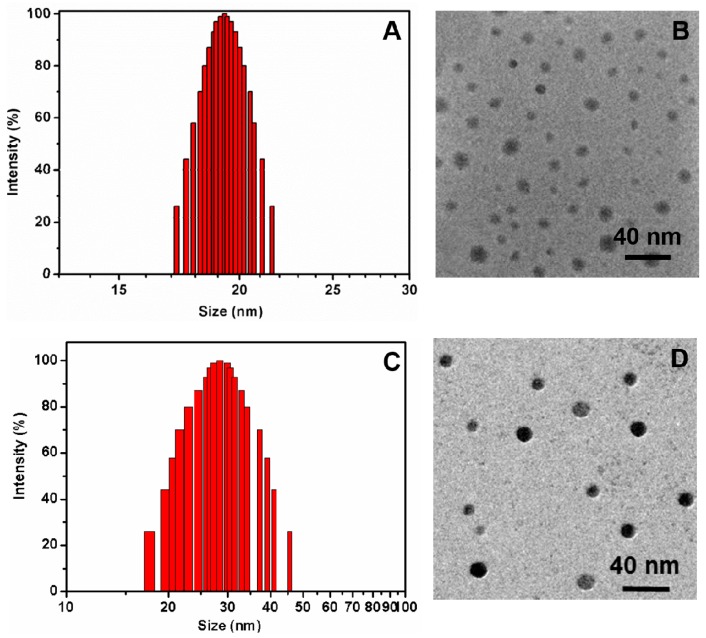
DLS plots (**A**,**C**) and TEM images (**B**,**D**, discrete and spherical blackspots) of blank (**A**,**B**) and IMQ-loaded (**C**,**D**) β-CD-(PLA-*b*-PDMAEMA-*b*-PEtOxMA)_21_ micelles.

Experimentally, the star block copolymer β-CD-(PLA-*b*-PDMAEMA-*b*-PEtOxMA)_21_ dissolved well in water. DLS measurement detected a single population of nanoparticles with an average hydrodynamic diameter *D*_h_ of approximately 19 nm and a narrow size distribution (PDI 0.218) (Figure 5A). Zeta potential value of the unimolecular micelles is 7.13 ± 1.35 mV. These nanoparticles remained stable in water without aggregation for as long as one month. TEM revealed that the star block copolymer formed discrete, largely-spherical unimolecular micelles with an average size of 15 nm (Figure 5B), slightly smaller than DLS measurement of hydrated particles [49,50]. Taken together, results from computer simulation and experiments point to the formation of unimolecular micelles in the aqueous solution.

### 3.4. IMQ Loading and pH-Responsive Release Kinetics

**Figure 6:**
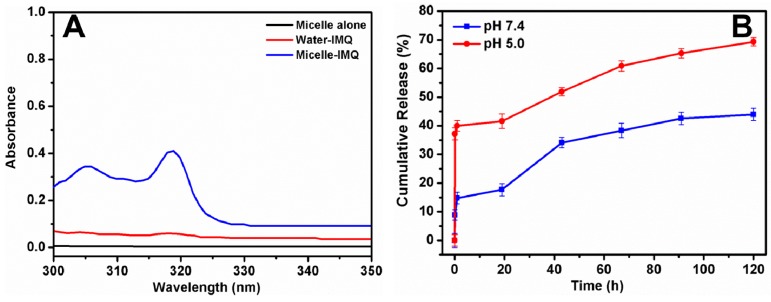
Enhancement of solubility and accelerated release of IMQ through encapsulation in β-CD-(PLA-*b*-PDMAEMA-*b*-PEtOxMA)_21_ unimolecular micelles. (**A**) UV spectra of the IMQ-loaded micelles solution, water-IMQ solution, and blank micelle solution, pH 7.4; (**B**) In vitro release profiles of IMQ from micelles at pH 7.4 and 5.0.

To evaluate the enhancement of IMQ solubility through micelle encapsulation, samples of free IMQ in water and IMQ loaded on micelles with equivalent IMQ content were analyzed. Blank micelles without IMQ did not absorb in this range of wavelength at all. Free IMQ showed insignificant absorbance in the range of 300–350 nm, due to low solubility in water at neutral pH (4.5 μg/mL) [21]. In contrast, IMQ loaded in micelles had higher adsorption in 300–330 nm with a prominent peak at 318 nm (Figure 6A), which the encapsulation in the unimolecular micelles enhanced the solubility of IMQ 12 fold, to 54 μg/mL, suggesting this unimolecular micelle system is better than the previously reported micelle system [21].

After IMQ loading, the size of the unimolecular micelles grew larger to an average diameter of 28 nm (by DLS) (Figure 5C) and 23 nm (by TEM), but the shape of the IMQ-loaded micelles was still round (Figure 5D). The increase of micelle size after drug loading was often explained by the extra volume needed for orderly packed polymer chains to accommodate drug molecules inside the micelles. It might also be due to drug-induced micelle aggregation. The amount of drug loading was 1.6 wt %. This value is slightly higher than a recently reported multimolecular polymer micelle system with PLA cores (1.2 wt %) [22]. Compared with multimolecular micelles, unimolecular micelles are generally expected to have smaller cores and, hence, lower encapsulation capacity. However, the PDMAEMA middle block of our star polymer might have contributed to higher drug loading observed here.

In vitro release kinetics of IMQ from the micelles was investigated. We showed in Figure 6A that IMQ had high absorbance at 318 nm when encapsulated in micelles, but when the drug was released into water, the absorbance at 318 nm was much reduced. This served as the basis for quantification of IMQ release from the hydrophilic core of the micelles into the hydrophilic environment of the water. When IMQ was still trapped in the micelles, UV absorbance at 318 nm was high. This was defined as 0% release. When all IMQ was released as free IMQ, UV absorbance at 318 nm was almost zero. This was defined as 100% release. As IMQ was released gradually, UV absorbance at 318 nm gradually decreased from the intensity of 0% release to 100% release. Thus, the time dependent reduction of IMQ absorbance at 318 was monitored at various time points until no further change occurred. Precisely because of this property of IMQ, we were able to quantify IMQ release into water more conveniently without having to separate free IMQ from IMQ-loaded micelles.

Figure 6B clearly showed that IMQ release from micelles was faster at slightly acidic pH. At pH 7.4, the release of IMQ was about 8% in 0.5 h, 15% in 1 h, and 44% after 120 h, owing to the slightly tight structure of the unimolecular micelles. The burst release including the first 0.5 h was found at this pH due to IMQ molecules located within the hydrophilic shell or at the interface between the hydrophilic shell and cationic middle layer, thus initial water penetration may have caused some IMQ to be released [51]. At pH 5.0, however, the release was about 35% in 0.5 h, 40% in 1 h and 70% after 120 h. This may be due to the fact that as the tertiary amine groups of the PDMAEMA block (pKa = 7.0) were protonated at pH 5.0, the reduction in hydrophobicity caused swelling of the micelles, and the entrapped IMQ could be released at an accelerated rate [52,53]. Moreover, the higher solubility of the IMQ in acidic medium (due to protonated amine in IMQ, pKa = 7.3) might have minor contribution to the faster release kinetics at pH 5.0. In addition, as reported in the literature with protonated amine-containing segments [54,55], we expect that the size of IMQ-loaded micelles with PDMAEMA block might become larger at acidic pH, which could be an another reason for the faster release. Based on these observations, it is reasonable to speculate that the IMQ-loaded pH-responsive unimolecular micelles with small particle size (<50 nm) and colloidal stability would be efficiently taken up by dendritic cells through multiple pathways, such as pinocytosis and phagocytosis, and subsequently release IMQ in the acidic endolysosome to engage in TLR7 signaling, as suggested by a previous report on acid-labile dextran microspheres [20]. To further reduce burst release in these polymer micelles, our future studies may use a longer pH-sensitive middle block or introduce crosslinks to stabilize the micelles.

### 3.5. Micelleplex Formation between Plasmid DNA and Star Polymer Micelles with and without IMQ

**Figure 7:**
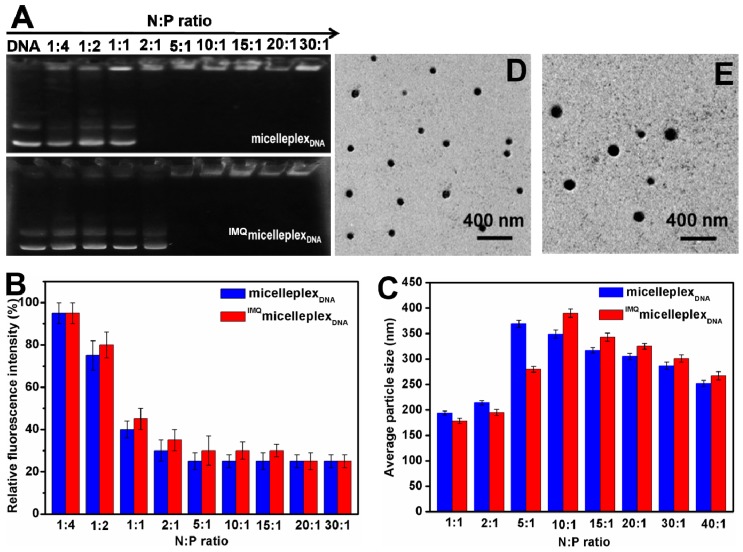
DNA binding, condensation by micelles, and size and morphology of micelleplexes with or without loading of IMQ. (**A**) Gel retardation assay; (**B**) ethidium bromide (EB) exclusion; (**C**) average particle size by DLS; TEM images of blank micelleplexes (**D**); and IMQ-loaded micelleplexes (**E**) at N:P = 20 (The discrete and spherical blackspots represented corresponding micelleplexes).

We hypothesize that the cationic PDMAEMA block of the star polymer should enable binding and condensation of plasmid DNA into micelleplexes. To assess DNA binding, a gel retardation assay was performed. Plasmid DNA migration through an agarose gel in the presence of blank micelles was completely retarded at N:P ratio of 2:1 or higher (Figure 7A). On the other hand, with IMQ-loaded micelles, complete retardation of plasmid DNA occurred at N:P ratios of 5:1 or higher. It is possible that IMQ encapsulation occupied part of the cationic PDMAEMA block, and, therefore, required a higher N:P ratio to achieve the same degree of DNA binding as the blank micelles. DNA condensation by micelles resulted in the exclusion of EB, a DNA-binding fluorescent dye, and quenching of its fluorescence. Both blank and IMQ-loaded micelles needed N:P ratios of at least 2:1–5:1 for maximal quenching of EB (Figure 7B). DLS experiments detected micelleplexes with average diameters ranging from 150–400 nm, depending on the N:P ratio, with PDI ranging from 0.1–0.4 (Figure 7C). There appeared to be a particle size maximum of 350–400 nm for blank and IMQ-loaded micelleplexes at N:P ratios of 5:1 and 10:1, respectively. A further increase in the N:P ratio led to smaller, presumably more tightly packed, micelleplexes. TEM of both blank and IMQ-loaded micelleplexes at N:P ratio of 20:1 showed round, discrete, nanoparticles having average size of 150–200 nm, with the IMQ-loaded micelleplexes being slightly larger (190 nm) than blank micelleplexes (155 nm) (Figure 7D,E). When dried for TEM, the micelleplexes appeared much smaller than those swollen in aqueous buffer as measured by DLS (300–350 nm).

Collectively, these results confirm the hypothesis that the unimolecular micelles were capable of binding and condensing plasmid DNA into discrete nano-scale micelleplexes. Having a hydrophilic outer shell of PEOxMA brushes did not prevent complexing with DNA, although it did require N:P ratio at least 5:1 or higher to form compact micelleplexes. Loading IMQ in the micelle core reduced only slightly the abilities of DNA binding and condensation, and it led to slightly larger micelleplexes than without the IMQ. Thus, it appears that combined loading of IMQ and plasmid DNA onto the same micelles is feasible without any mutual interference, likely because the two cargos are spatially segregated—with the IMQ partitioning into the hydrophobic core and the plasmid DNA interacting with the cationic middle segment.

### 3.6. Transfection of DCs by Micelleplexes with and without IMQ

**Figure 8:**
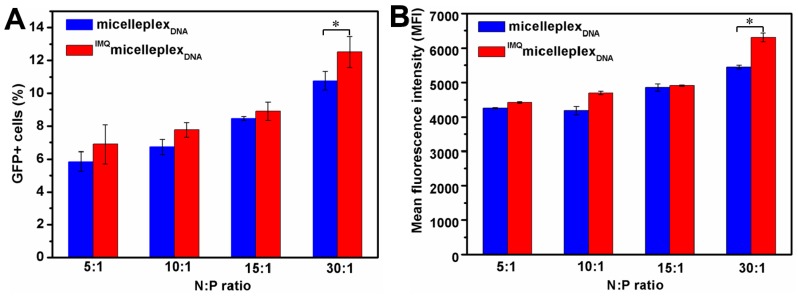
Transfection efficiency of DC2.4 cells by micelleplexes determined by flow cytometry. Quantification by the percentage of transfected GFP^+^ cells (**A**) and mean fluorescence intensity (**B**). * *p* < 0.05.

Transfection efficiency of the micelleplexes was assessed in mouse dendritic cell line (DC2.4) with GFP as a reporter. Transfection was conducted for 4 h in cell media without serum and expression of GFP quantified by flow cytometry after 20 h of culture with serum-containing media. Micelleplexes were prepared using N:P ratios of 5:1 through 30:1, either with or without loading of IMQ. A cytotoxicity assay showed that micelleplexes with N:P ratios of up to 20:1 maintained greater than 90% cell viability with the exception of N:P ratio of 30:1 (70% viability) (Figure S1). Special care was taken to avoid false positive signals of GFP positive cells due to potential changes in cell autofluorescence after treatment with the polymer. Figure 8A showed that 6%–14% DCs were transfected by micelleplexes. Higher N:P ratios resulted in higher percentages of GFP^+^ cells and higher MFI. IMQ loading did not significantly alter the transfection efficiency, with the exception of the N:P ratio of 30:1, which resulted in statistically significant (*p* < 0.05) increases of the fraction of GFP^+^ cells and MFI (Figure 8B). This finding confirms that encapsulating IMQ in the hydrophobic core of the micelles has no negative impact on the function of the micelles as gene carriers, consistent with our other observations that IMQ-loading caused only minor changes in DNA binding, condensation, and micelleplex size and morphology. It also implies that, within the time frame of the experiment (24 h), IMQ and TLR7 signaling have not produced any biological changes in the cells that might impede intracellular gene delivery processes mediated by this particular amphiphilic star block copolymer. Whether this conclusion holds true for other polymeric gene carriers with different chemical composition and architecture is a question that requires further study. Moreover, the importance of dosing and release kinetics of IMQ co-delivered with plasmid DNA in stimulating DC maturation and antigen expression will be investigated in the future.

## 4. Conclusions

An amphiphilic pH-responsive star block copolymer has been designed to serve as a nano-scale vehicle for combined loading and delivery of IMQ and plasmid DNA. Living polymerization methods (ARGET ATRP and ROP) were used to precisely control the chain length and composition of the star polymer. The polymer formed small, narrowly-dispersed unimolecular micelles in an aqueous buffer. Solubility of IMQ had been much enhanced by encapsulating in the PLA core of the micelle. The release of IMQ could be accelerated at mildly acidic pH, owing to the pH-responsive protonation of the PDMAEMA block of the micelles. These micelles were also capable of forming nano-scale micelleplexes with plasmid DNA and transfecting dendritic cells in vitro. IMQ-loading had a moderate impact on the formation and size of micelleplexs, but the gene transfection capacity of the micelleplexes remained intact. With further optimization, this well-defined pH-responsive star block copolymer, with its dual capacities of loading IMQ and plasmid DNA, could be useful nano-carriers for potential synergistic delivery of gene-based immunotherapeutics and vaccines.

## Supplementary Materials

The following are available online at www.mdpi.com/2073-4360/8/11/397/s1, Figure S1: In vitro cytotoxicity of micelleplexes after 24 h incubation at different N:P ratios determined by MTT assay against DC 2.4 cells (*n* = 6).
