# Supplementary material for: Co-Delivery of Imiquimod and Plasmid DNA via an Amphiphilic pH-Responsive Star Polymer that Forms Unimolecular Micelles in Water
Source: Polymers (Basel). 2016 Nov 16;8(11):397. doi: 10.3390/polym8110397 (PMC6431966; doi:10.3390/polym8110397)
Supplement: Supplementary file 1 [file polymers-08-00397-s001.pdf]

Supplementary Materials: Co-Delivery of Imiquimod and Plasmid DNA via an Amphiphilic pH-Responsive Star Polymer that Forms Unimolecular Micelles in Water

Wenjing Lin, Na Yao, Hongru Li, Samuel Hanson, Wenqing Han, Chun Wang and Lijuan Zhang

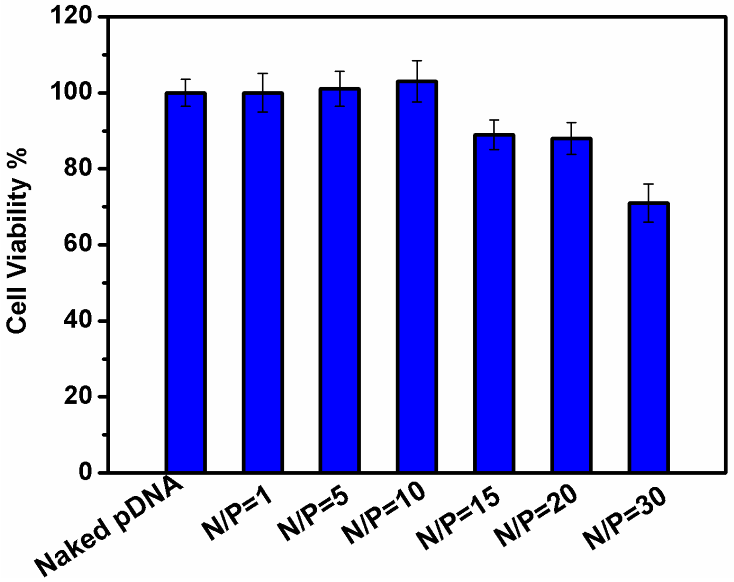

Figure S1. In vitro cytotoxicity of micelleplexes after 24 h incubation at different N:P ratios determined by MTT assay against DC 2.4 cells (n = 6).
